# Microsymbiont diversity and phylogeny of native bradyrhizobia associated with soybean (*Glycine max* L. Merr.) nodulation in South African soils^☆^

**DOI:** 10.1016/j.syapm.2016.05.009

**Published:** 2016-07

**Authors:** Judith Naamala, Sanjay K. Jaiswal, Felix D. Dakora

**Affiliations:** a Department of Crop Sciences, Tshwane University of Technology, Pretoria, South Africa; b Department of Chemistry Tshwane, University of Technology, Arcadia Campus, Pretoria, South Africa

**Keywords:** Colony morphology, 16S rDNA-RFLP, Horizontal gene transfer, Housekeeping genes

## Abstract

The genetic diversity and identification of slow- and fast-growing soybean root nodule bacterial isolates from different agro-climatic regions in Mpumalanga, Limpopo and Gauteng Provinces of South Africa were evaluated. The 16S-rDNA-RFLP analysis of 100 rhizobial isolates and eight reference type strains placed the isolates into six major clusters, and revealed their site-dependent genomic diversity. Sequence analysis of single and concatenated housekeeping genes (*atpD*, *glnII* and *gyrB*), as well as the symbiotic gene *nifH* captured a considerably higher level of genetic diversity and indicated the dominance of *Bradyrhizobium diazoefficiens* and *Bradyrhizobium japonicum* in Mpumalanga, Limpopo and Gauteng Provinces. Gene sequence similarities of isolates with type strains of *Bradyrhizobium* ranged from 97.3 to 100% for the 16S rDNA, and 83.4 to 100% for the housekeeping genes. The *gln*II gene phylogeny showed discordance with the other genes, suggesting lateral gene transfer or recombination events. Concatenated gene sequence analysis showed that most of the isolates did not align with known type strains and might represent new species from South Africa. This underscores the high genetic variability associated with soybean *Bradyrhizobium* in South African soils, and the presence of an important reservoir of novel soybean-nodulating bradyrhizobia in the country. In this study, the grouping of isolates was influenced by site origin, with Group I isolates originating from Limpopo Province and Groups II and III from Mpumlanga Province in the 16S rDNA-RFLP analysis.

## Introduction

Soybean (*Glycine max* [L.] Merrill) is a grain legume belonging to the family Leguminosae (Fabaceae) and sub-family Papilionoideae [16], [23], [65]. It originated from North-eastern China and is currently cultivated worldwide under various climatic conditions [6], [46]. It may have been introduced to Africa in the 19th century by Chinese traders along the east coast of Africa [50]. Its mature grain contains about 40% protein, 30% carbohydrate (cellulose, pectin and phytic acid), 10% digestible fibre, the vitamins E, K, riboflavin, thiamine, niacin and choline and minerals such as K, Mg, Ca, Zn, Fe and Cu, as well as anti-oxidants [16], [37]. Soybean alone accounts for 80% of the land area used for legume cultivation in the world and about 68% of the world's legume production [23]. About 109 980 000 ha of land are under soybean production worldwide [59]. However, soybean production in Africa is constrained by several factors, including the lack of compatible rhizobia in many African soils [41]. Promiscuous soybean varieties, which nodulate with indigenous soil rhizobia, have been bred to overcome the nodulation problem [1], [41].

Diverse groups of bacteria (fast- and slow-growers) belonging to the genera *Bradyrhizobium*, *Sinorhizobium* (*Ensifer*) and *Mesorhizobium* are responsible for establishing effective N_2_-fixing symbiosis with soybean [57], [62]. The slow-growers are distributed across different species, which include *Bradyrhizobium japonicum* [28], *Bradyrhizobium elkanii* [31], *Bradyrhizobium liaoningense* [68], and *Bradyrhizobium yuanmingense* [70]. The fast-growers consists of *Sinorhizobium fredii* and *Sinorhizobium xinjiangense* [12], [24], [29], [44], while *Mesorhizobium tianshanese* [11], with varying generation time, has also been reported to be a soybean microsymbiont. The *B. japonicum* and *B. elkanii* species have been found in various climatic regions across the world, while *B. liaoningense*, *B. yuanmingense* and *Bradyrhizobium canariense* have not yet been surveyed worldwide. The alkaline soils of temperate to subtropical climates of South and South-east Asia are home to *B. liaoningense* [2], [6], [22], [33], while the warm tropical climates of India, Kenya and Nepal favour *B. yuanmingense* [6], [46], [64]. The acid-tolerant *B. canariense* was found in North-east China [69]. In contrast, the fast- growing *S. fredii* and *S. xinjiangense* species were isolated from saline-alkaline soils in China, Vietnam, and Japan [7], [22], [33], [35], [44], [54]. Species of *M. tianshanense* were also isolated from China [11].

Characterisation and classification of rhizobia into different groups has been achieved using phenotypic, physiological and/or molecular methods. Phenotypic characterisation deals with traits exhibited by the rhizobial colonies, which include colony shape, colour and size [67]. Molecular characterisation, on the other hand, involves studying genome of the rhizobial cell. The molecular techniques commonly used to study rhizobial diversity include restriction fragment length polymorphism (RFLP), amplified fragment length polymorphism (AFLP) and rapid amplified polymorphic DNA (RAPD) [9], [32], [34]. Other methods such as DNA–DNA hybridisation, sequence analysis of the 16S rDNA gene and multilocus sequence analysis (MLSA) have also been used to study rhizobial taxonomy and diversity [15], [36], [39].

MLSA involves the study of sequences of the more conserved housekeeping genes such as *atpD*, *glnII* and *gyrB* genes. The sequencing of the 16S rDNA gene, on the other hand, is accurate at the genus level but poor at inter- and intra-species discrimination due to its low variation in sequences [36], [60]. MLSA is currently considered a more reliable alternative for studying genomic relationships between rhizobia [42], [58]. The genes used in MLSA have a higher degree of sequence divergence, hence MLSA is good for inter- and intra-species identification [36]. Additionally, housekeeping genes are more discriminatory and can therefore identify rhizobial strains from closely related lineages [36]. The housekeeping genes analysed in this study were reported previously as good taxonomic markers [36], [72].

Despite the growing cultivation of soybean in South Africa, and Africa at large, there is very limited information in the literature on the type of rhizobia nodulating this legume crop in South African soils. Studies of the type of rhizobia preferred by the soybean in South Africa, the dominant rhizobial strains in local soils, and their genetic diversity are needed to serve as guide for the production of soybean inoculants. The aim of this study was to determine the genetic diversity and phylogenetic identification of bacteria nodulating soybean in the different agro-climatic regions of South Africa.

## Materials and methods

### Sampling of root nodules and soils for rhizobial isolation

Two sources of root-nodule bacteria were used in this study: i) rhizobia isolated from soybean nodules collected from inoculated and uninoculated farmers’ fields in different areas of Mpumalanga Province in South Africa, and ii) rhizobia trapped in pot experiments using surface-sterilised soybean seed and uncultivated soils (10–20 cm depth) collected from different locations in Mpumalanga, Gauteng and Limpopo Provinces. The sites of nodule and soil collection are indicated in Table S1. Soil samples were processed and used to trap rhizobia, as described by Somasegaran and Hoben [51].

### Rhizobia isolation and morpho-physiological characterisation

Rhizobia from soybean root nodules were isolated following standard procedures [61], [67]. Colony morphology and acid/alkaline reactions were evaluated on YMA containing bromothymol blue (25 μg/ml) as indicator. Pure single colony of each isolate was used for host-nodulation test with soybean variety PAN 1666, in fulfilment of Koch's postulate. Reference strains such as *Rhizobium leguminosarum* USDA 2370^T^, *B. japonicum* USDA 6^T^, *B. diazoefficiens* USDA 110^T^, *B. elkanii* USDA 76^T^, *Ensifer meliloti* (*Sinorhizobium meliloti*) USDA 1002^T^ and *Ensifer medicae* (*S. medicae*) USDA 103^T^ used in this study, were obtained from the National *Rhizobium* Germplasm Resource Collection USDA-ARS-SGIL, Beltsvile, USA.

### Rhizobial DNA isolation and PCR amplification of 16S rDNA gene region

Rhizobial genomic DNA was extracted using GenElute Bacterial Genomic DNA kit according to the manufacturer's instructions (Sigma Aldrich, USA) and examined using 1% agarose gel containing ethidium bromide. The 16S-rDNA regions of bacterial genomic DNA was amplified in 25 μL reaction mixture containing 3 μl (5×) My Taq PCR buffer, 0.1 μl Taq polymerase (5 U) (Bioline, USA), 1 μl (10 pM) of each forward and reverse primer and 1 μL (40–50 ng) DNA as template. DNA amplifications were performed by Thermal cycle (T100 BIORAD, USA) with respective primers and standard temperature profile and examined on horizontal gel electrophoresis in 1.2% agarose gel (Table S2 [43], [52]).

### Restriction fragment length polymorphism (RFLP) analysis of PCR-amplified 16S rDNA region

Products of the PCR-amplified 16S rDNA region were digested with three different four base-cutting restriction enzymes (namely, MspI, RsaI and HaeIII), following the procedure recommended by the manufacturer (Thermo Scientific, Lithuania) and digested products were separated by electrophoresis in 2.5% agarose gel.

### RFLP cluster analysis of 16S rDNA region

The digested fragments of restriction enzymes were scored as “1” for presence, and “0” for absence of homologous bands. A dendrogram was constructed from the distance matrix using the un-weighted pair group method with arithmetic mean algorithm (UPGMA) with the help of NTSYSpc 2.1 software [47]. The Shannon–Wiener diversity index (*H*′) [49] was estimated based on the number of isolates of each province belonging to each cluster of the 16S rDNA-RFLP dendrogram.

### PCR amplification, sequencing and phylogenetic analysis of housekeeping (*atpD*, *glnII* and *gyrB*) and symbiotic (*nifH*) genes

The PCR amplification of *atpD*, *glnII*, *gyrB* and *nifH* genes of rhizobial genome was done as described above for 16S rDNA. The primers used and thermal cycle conditions are listed in Table S2. The PCR amplified products of 16S rDNA, symbiotic gene (*nifH*) and housekeeping loci (*atpD*, *glnll* and *gyrB*) were purified using GeneJET PCR purification kit (Thermo Scientific, Lithuania). The purified samples were sequenced (Macrogen, Netherlands), and the quality of all sequences checked using BioEdit 7.0.0 software [21]. NCBI GenBank databases were used to identify species that were closely related to our test strains by means of the BLAST_n_ program, and sequences submitted to the NCBI GenBank to get accession numbers (Table S3). Reference type sequences were selected to align with sequences of the test strains using MUSCLE [17] for construction of phylogenetic trees using MEGA 6.0 program [56]. Phylogenetic trees were generated by kimura 2-parameter method to calculate evolutionary distances [30], and evolutionary history was inferred using the Maximum-Likelihood method with 1000 bootstrap support [18]. Nucleotide information was obtained from conserved, variable, parsimony-informative, and singleton regions using consensus sequences. Tajima neutral mutation test was done by pair-wise comparison of analysis of the genetic variation by observing the number of nucleotide differences. Codon positions included were 1st + 2nd + 3rd + noncoding. All positions containing gaps and missing data were eliminated.

## Results

A total of 110 pure single-colony isolates were obtained and tested for plant nodulation under glasshouse conditions in conformity with Koch's postulate. Out of the 110 test isolates, 100 were identified as soybean rhizobia, given their ability to nodulate non-promiscuous soybean variety PAN1666 in plant nodulation assays.

Colonies of soybean nodule isolates appeared on YMA plates over variable time periods (2–14 days), exhibited different colony shapes, and failed to absorb the red colour of Congo red dye on YMA plates (Table S4). Isolates TUTMP16.2, TUTMP14.10.1, TUTMP14.10.3, TUTMP14.10.3b, TUTGP10.10.1, TUTLI1.10.3, TUTLI2.20.7, TUTLI4.20.5, TUTLI4.20.16, TUTLI8.20.1, TUTLI8.20.5 and TUTLI8.20.5b formed colonies before 4 days after inoculation on YMA plates. PCR amplification of 16S rDNA gene region of each isolate's genome produced a single band of approximately 1500 bp. The isolates exhibited variation in the banding pattern of the 16S rDNA gene when digested with 4-base cutter MspI, RsaI and HaeIII restriction endonucleases. All tested restriction endonucleases were able to produce polymorphic banding pattern with the test isolates. Restriction endonucleases MspI, RsaI and HaeIII yielded 16(A-P), 12(A-L) and 19 (A-S) restriction banding patterns, respectively (Table S4). On the basis of combined digested banding patterns, the dendrogram clustered isolates and reference strains into six main groups by the binary matrix 0/1 (0 for absence and 1 for presence of the restriction type (see Fig. S1). Group I consisted of 15 rhizobial isolates with a similarity coefficient of 0.22. No reference strain clustered with this group. Group II consisted of 22 rhizobial isolates that clustered with reference *Bradyrhizobium* strains USDA 6, H1, USDA 76 and USDA 110 with a similarity coefficient of 0.20. Groups III and IV respectively consisted of 20 and eight rhizobial isolates with a similarity coefficient of 0.12 and 0.08. No reference strain clustered with these groups. Group V had two rhizobial isolates (7 and 8) with a similarity coefficient of 0.50 and clustered with reference strains *Rhizobium leguminosarum* (USDA 2370), *Sinorhizobium meliloti* (USDA 1002) and *Sinorhizobium medicae* (USDA 1037). Group VI was the largest cluster with 31 isolates from Mpumalanga and Limpopo Provinces. Two rhizobial isolates (namely, 58 and 70) did not cluster with any reference strain or other test isolates, and stood independently with a 0.00 similarity coefficient to the rest of the isolates.

The Shannon–Weiner diversity index was highest (1.47) in Mpumalanga Province, followed by Limpopo (1.37), and then Gauteng Province (0.69) (Table S1).

### Phylogenetic analysis of 16S rDNA, *nifH*, *atpD*, *gyrB* and *glnII* genes

A total of 22 bradyrhizobial isolates were selected from 16S rDNA-RFLP analysis as representative of each cluster to get the phylogenetic position. The PCR amplification of *nifH* (dinitrogenase reductase), and housekeeping genes [*atpD* (ATP synthase beta chain), *gyrB* (DNA gyrase) and *glnII* (glutamine synthase II)] yielded a single band of about 800 bp for *nifH*, *atpD* and *gyrB*, and 750 bp for *glnII* products.

Based on partial 16S rDNA gene sequence comparisons with the NCBI GenBank references, all the tested isolates were identified as *B. diazoefficiens*, *B. japonicum, B. liaoningense* and *B. yuanmingense.* The phylogenetic tree of the 16S rDNA gene grouped isolates into four distinct clusters (I–IV). Isolates TUTLl2.20.6, TUTLl4.10.7, TUTLl2.20.7, TUTLl4.10.9, TUTMP7.7, TUTMP2.9 and TUTMP9.7 grouped with reference strains of *B. liaoningense, B. yuanmingense* and *B. japonicum* in Cluster I with all isolates having 97–100% sequence identity with *Bradyrhizobium* type strains. Isolates TUTGP10.10.2, TUTMP18.11 and TUTMP18.6 aligned with *B. diazoefficiens* USDA 110 in Cluster II with a low bootstrap support of 60%. Only isolate TUTLl2.10.7 grouped with *Bradyrhizobium iriomotense, Bradyrhizobium huanghuaihaiense, Bradyrhizobium denitrificans* and *Bradyrizobium ganzhouense* in Cluster III. Surprisingly, isolate TUTMP16.11 clustered with *Rhizobium lusitanum* P1-7 with a high 99% bootstrap support and with sequence identity of 99.1%, while showing 91.0–92.2% identity with a type strain of *Bradyrhizobium* (tree not shown).

### Analysis of individual housekeeping genes

Sequence analysis of the housekeeping genes *atpD*, *glnII* and *gyrB* respectively revealed 83.4–100%, 88.4–100% and 89.9–100% similarities with type strains of *Bradyrhizobium*. The lowest level of conservation (54.57%), the highest variable (45.43%) and the highest singleton sequences (19.25%) were observed for *gyrB*. The levels of conservation were 65.26% for *atpD* and 61.74% for *glnII*, while the highest parsimony-informative sequence information (32.21%) was observed in *glnII* (Table S5). In Tajima neutral mutation test, the highest (208) segregation sites was observed in *gyrB* locus, with *atpD* and *gyrB* showing negative D values (Table S6).

Phylogenetic trees were constructed using the maximum likelihood method for each housekeeping gene and this resulted in different *Bradyrhizobium* groupings. Isolates that were not clearly delineated at genus or species level by 16S rDNA gene, were distinctly separated into *B. elkanii, B. japonicum, B. yuanmingense*, *B. iriomotense* and *B. diazoefficiens* in the housekeeping gene phylogeny. Isolates with difficulties in PCR amplification or poor sequence quality were excluded in tree construction. In each of the housekeeping gene's phylogenetic tree, most of the test isolates were consistently grouped into a major clade with *B. diazoefficiens* (*atpD* and *gyrB*) and *B. elkanii* (*glnII*).

**Fig. 1:**
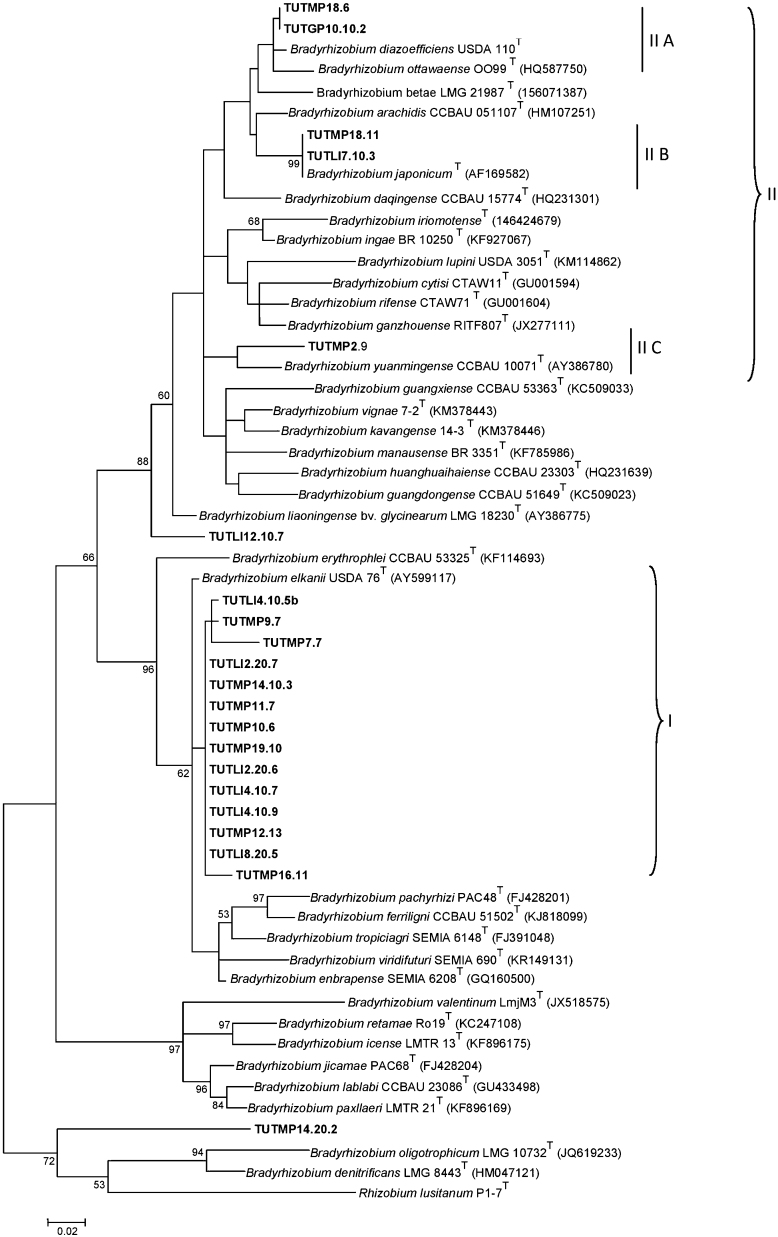
Phylogenetic tree based on *glnII* sequences generated by Maximum-Likelihood method. Bootstrap values (1000 replicates) are indicated above the branches.

The phylogenetic tree from the *atpD*, *glnII* and *gyrB* gene analysis formed different clusters with *B. diazoefficiens*, *B. japonicum*, *Bradyrhizobium jicamae*, *B. yuanmingense* and *B. elkanii* (Figs. 1, S2 and S3). The single gene phylogenetic study showed incongruency of the isolates with similar type strains in all studied phylograms. Isolates TUTLl4.10.7, TUTMP18.11, TUTMP16.11, TUTMP14.10.3 and TUTLl4.10.5b showed similar species level cluster with *B. diazoefficiens* in *atpD* and *gyrB* phylograms, but aligned with *B. elkanii* in *gln*II phylogeny. There was a major shift of the isolates between clusters, with the relative positions of a few strains varying between *atpD*, *glnII* and *gyrB* genes trees.

All isolates from Limpopo Province, (except for isolate TUTLl2.20.7) clustered with *B. diazoefficiens* and *B. japonicum* in *atpD* and *gyrB* phylogenies, while Mpumlanga isolates clustered with *B. diazoefficiens, B. japonicum, Bradyrhizobium guangxiense, Bradyrhizobium guangdongense, B. yuanmingense, B. jicamae* and *B. elkanii* (Figs. S2 and S3). Isolate TUTGP10.10.2 from Gauteng Province also grouped with *B. diazoefficiens* in *gln*II and 16S rDNA phylograms.

Reconstruction of the phylogenetic tree of *glnII* sequences revealed that all isolates were grouped into two major clusters (I, II). Unlike *atpD* and *gyrB* phylogenetic analysis, most of the isolates proximally related with *B. elkanii* in Cluster I (Fig. 1), while Cluster II was divided into three subclusters (IIA, IIB, IIC). Subcluster IIA had isolates TUTMP18.6 and TUTGP10.10.2 which delineated with *B. diazoefficiens*, whereas isolates from subcluster IIB and IIC grouped with *B. japonicum* and *B. yaunmingense*, respectively (see Fig. 1). Isolate TUTLl2.10.7 distantly linked with Cluster II as an outgroup, while TUTMP14.20.2 stood alone with *Bradyrhizobium oligotrophicum* and *B. denitrificans* type strains (Fig. 1).

### Phylogenetic analysis based on the combined *atpD* and *gyrB* gene sequences

**Fig. 2:**
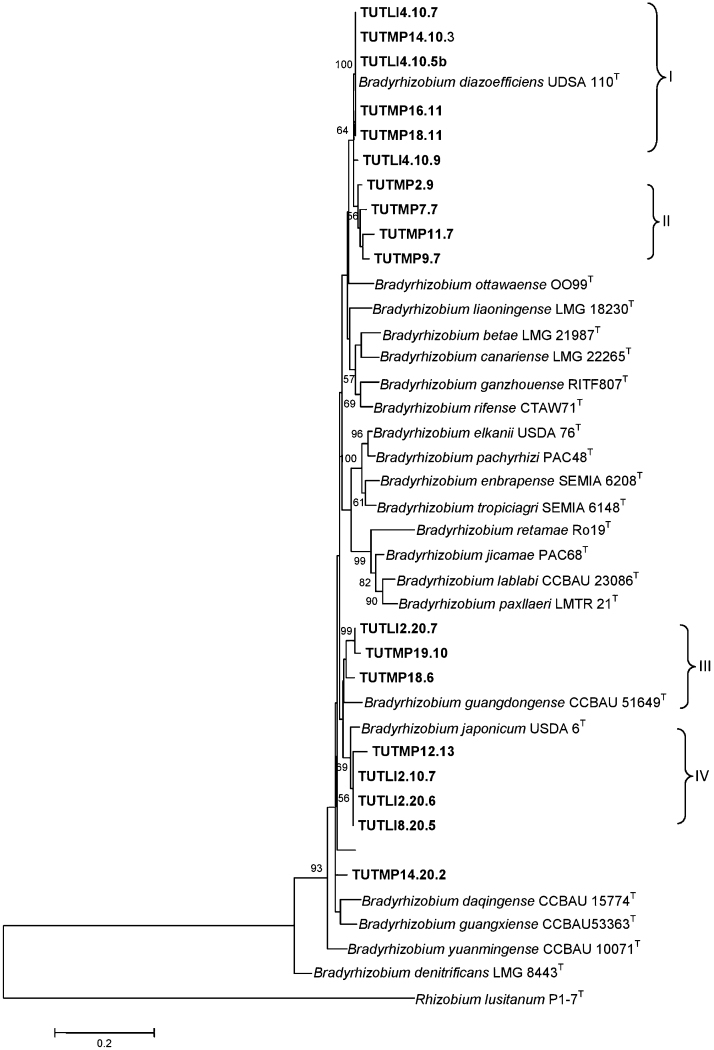
Phylogenetic tree based on *atpD* + *gyrB* sequences generated by Maximum-Likelihood method. Bootstrap values (1000 replicates) are indicated above the branches.

A phylogenetic tree based on concatenated genes confirmed the presence of diverse and novel type of *Bradyrhizobium* isolates nodulating soybean in South Africa. With the discordance observed in test isolates from *glnII* and 16S rDNA phylograms in comparison to those of *atpD* and *gyrB* housekeeping genes, we constructed concatenated tree with *atpD* + *gyrB* sequences (Fig. 2). The concatenated phylogeny was based on average consensus sequences of 843 analysed sites containing 509 (60.6%), 331 (39.4%), 170 (20.24%) and 161 (19.17%) of conserved, variable, parsimony-informative and singleton information sites, respectively (Table S5). The constructed concatenated tree revealed four large clusters (Clusters I–IV) with *Bradyrhizobium* species corresponding to *B. diazoefficiens*, *Bradyrhizobium guangdongense* and *B. japonicum* (Fig. 2). Five test strains in Cluster I grouped with *B. diazoefficiens* with high bootstrap support (100%) and 100% sequence identity, while isolate TUTLl4.10.9 was linked to the Cluster I outgroup with 64% bootstrap support and 99.1% sequence identity (Fig. 2; Table S7). In the *atpD* phylogram, the same isolate TUTLl4.10.9 grouped with *B. japonicum* in Cluster II.

Cluster II consisted of four isolates that failed to group with any type strains even though they were aligned with *B. diazoefficiens* in the *gyrB* phylogenetic tree. Although the four isolates converged in Cluster II in the concatenated phylogeny, they behaved differently in individual gene phylogenies. In the *atpD* tree, TUTMP2.9 grouped with *B. yuanmingense*, isolate TUTMP11.7 aligned with *B. elkanii*, TUTMP7.7 closed to *B. jicamae*, while isolate TUTMP9.7 stood alone. Monophyletic Cluster IV also had four isolates (TUTMP12.13, TUTLl2.20.6, TUTLl2.10.7 and TUTLl8.20.5) that grouped with *B. japonicum* in the same way as in the *gyrB* phylogram with a 69% bootstrap support and 97.1 to 98.3% sequence identity. Cluster III comprised three isolates that grouped without any reference type strains, but close to *B. guangdongense*, while isolate TUTMP14.20.2 stood alone in the tree (Fig. 2).

### Phylogenetic analysis based on the *nifH* gene sequence

**Fig. 3:**
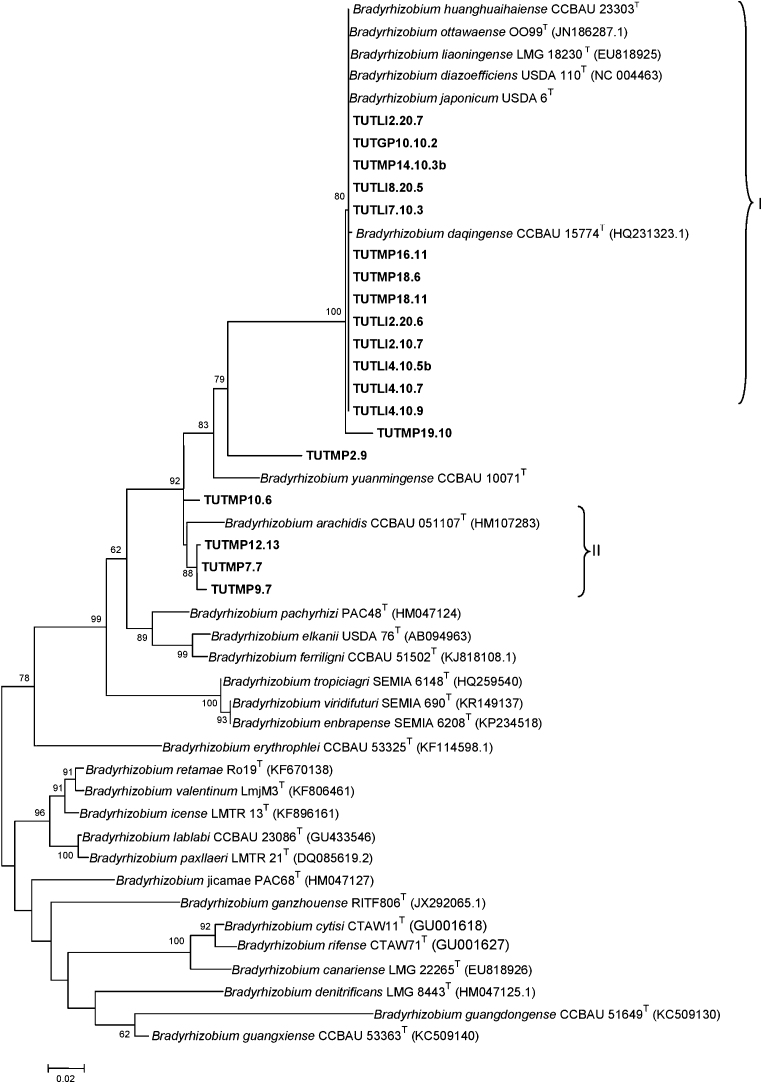
Phylogenetic tree based on *nifH* sequences generated by Maximum-Likelihood method. Bootstrap values (1000 replicates) are indicated above the branches.

The reconstructed phylogenetic tree based on *nifH* was congruent with the tree built with *atpD* gene. Isolates TUTMP11.7, TUTMP14.20.2 and TUTMP14.10.3 were not included in the *nifH* study due to poor sequence results. In the *nifH* phylogenetic tree, all isolates from Limpopo Province, isolate TUTGP.10.10.2 from Gauteng, and some isolates from Mpumalanga were tightly clustered in one clade along with type strains such as *Bradyrhizobium daqingense* CCBAU 15774^T^, *Bradyrhizobium ottawaense* OO99^T^, *Bradyrhizobium huanghuaihaiense* CCBAU 23303^T^, *B. liaoningense* LMG 18230^T^, *B. japonicum* USDA 6^T^ and *B. diazoefficiens* USDA 110^T^ with a high 80 bootstrap support and 100% pair-wise sequence identity (Fig. 3). Isolates TUTMP2.9 and TUTMP19.10 were located on the outgroups of Cluster I with bootstrap support 79 and 100, respectively, and pairwise sequence similarity of 91.6% and 98.3% with the *Bradyrhizobium* type strains present in Cluster I. Cluster II consisted of only Mpumalanga isolates TUTMP10.6, TUTMP9.7, TUTMP7.7 and TUTMP12.13 without any type or reference strains (Fig. 3). In the *atpD* phylogram (Fig. S2), isolate TUTMP10.6 was closely grouped with *B. elkanii* USDA 76 with a 56% bootstrap support, while TUTMP12.13, TUTMP9.7 and TUTMP7.7 clustered together without any reference strains.

## Discussion

### Phenotypic variation among bacterial symbionts nodulating soybean

In this study, we analysed rhizobial isolates from soybean root nodules obtained from different agro-climatic zones in South Africa. Out of 110 isolates, 100 formed root nodules with soybean variety PAN 1666 in fulfilment of Koch's postulate. Of the 100 rhizobial isolates, 66 were trapped from soils that had no recent history of crop cultivation, while 34 were isolated from farmers’ fields, previously inoculated with *B. japonicum* inoculants. Although it has been reported that some N_2_-fixing isolates can lose their symbiotic genes and thus fail to nodulate the homologous host during nodulation assay [5], [25], that was not the case in this study. The 100 bacterial symbionts comprised both fast- and slow-growers, and respectively produced acid and alkali reaction on BTB-containing YMA plates (Table S4).

Soybean-nodulating bacteria in the genus *Bradyrhizobium* are usually slow-growers (growth rate of ≥5 days) and alkali-producing with smaller colonies (<2 mm). However, strains from the genus *Sinorhizobium*, which also nodulates soybean, are fast-growers (growth rate of ≤5 days), and acid-producing with larger colonies (>2 mm) [51], [61]. The data in Table S4 clearly show that soybean-nodulating isolates from South Africa exhibited a huge diversity, as evidenced by the proportions of colony characteristics which included 87% dome shape, 5% conical, 6% flat and 2% oval isolates. Various studies [3], [13], [38] have similarly found isolate diversity in relation to cell growth rate (82% slow-growers vs. 18% fast-growers), as well as bromothymol blue reaction (83% alkali and 17% acid-producing). Some fast-growers (TUTMP15.3 and TUTGP10.10.1) showed alkali reaction, while slow-growers (TUTMP5.16, TUTMP11.1 and TUTMP19.10) showed acid reaction with BTB on YMA plates.

### Molecular analysis of microsymbiont diversity of soybean-nodulating bacteria

PCR-RFLP has become a potent tool for classifying bradyrhizobial isolates due to the restriction site variation in certain amplified regions of the genome of different rhizobia [26], [40]. Soybean is nodulated by diverse bacterial symbionts that can undergo differentiation, resulting in new rhizobial types [1], [60], [69]. In this study, 16S rDNA-RFLP was used to molecularly characterise bradyrhizobial isolates from soybean root nodules. Based on the patterns of digested fragments, the number and distance of restriction sites recognised by a particular restriction endonuclease were found to differ among the different rhizobial isolates [4]. In fact, some of the isolates showed similar fingerprint after restriction digestion. This supported the findings of Amarger [4] which showed that similar banding pattern of bacterial isolates, when separately digested by more than two restriction enzymes, is an indication of genetic relatedness.

Some test isolates also showed variable RFLP digestion profiles of the 16S rDNA genomic region, which confirmed the presence of diverse bradyrhizobial isolates in the local microsymbiont population. This is inconsistent with the findings of Germano et al. [19] which found no diversity reflection in the16S rDNA sequence region of rhizobia. Furthermore, a dendrogram constructed from the RFLP digestion profiles clustered the bacterial isolates into six major groups. Except for the isolates in Group II, the RFLP patterns of the isolates in Groups I, III, IV and VI were clearly different and showed that they are genetically distinct from all the reference strains. However, Group II isolates were highly related to reference strains *B. japonicum* (USDA 6^T^, H1), *B. diazoefficiens* (USDA 11  ^T^), and *B. elkanii* (USDA 76^T^), a finding consistent with the RFLP results of Abaidoo et al. [1]. In other previous studies, RFLP-PCR analysis of the 16S rRNA gene clearly differentiated *Bradyrhizobium* from *Rhizobium*, and even *B. japonicum* from *B. elkanii* [19], [32], [63]. In this study, the grouping of isolates was influenced by the site of origin. For example, the isolates in Group I originated from Limpopo Province, while Group III and IV were isolated from Mpumalanga Province. Our finding contrasted those of Yang & Zhou [69] and Grossman et al. [20], who showed that the clustering of soybean isolates, in their studies, was not influenced by the site of origin.

In this study, the phylogeny of soybean rhizobia was studied using sequence analysis of the 16S rDNA, *nifH*, *atpD*, *glnII* and *gyrB* genes. Based on the 16S rDNA gene analysis, the estimated similarity percentage between test isolates and type reference strains of *Bradyrhizobium* from NCBI GenBank ranged from 97.3 to 100%, except for isolate TUTMP16.11, which showed 89.8–92.2% identity. Thus, even though the 16S rDNA gene is highly conserved in members of the genus *Bradyrhizobium* [39], [66], our analysis showed that many isolates stood alone in a branch separate from other species of *Bradyrhizobium* and also revealed incongruency with studied housekeeping genes, a finding inconsistent with the results of Zilli et al. [74].

In this study, considerable phylogenetic congruency was found with the sequencing of *atpD* and *gyrB* genes, but not *glnII*, which produced discordant results. The observed phylogenetic incongruences could be attributed to the different evolutionary histories of the genes studied [73], intra-genomic re-arrangements and vertical/horizontal gene transfer, as well as subsequent recombinations that probably occurred in the genes [10], [14], [27], [45], [48], [71]. Gene recombination is known to play a major role in the evolution of soybean rhizobia. Li et al. [33] have reported significant gene recombination in chromosomal housekeeping genes of soybean rhizobia, just as they also observed both lateral and vertical gene transfer among soybean-nodulating microsymbionts. Symbiotic genes such as the *nifH* gene are easily transmitted through genetic re-arrangement [53].

In the Tajima neutral mutation test, the D values for *glnII* and *nifH* were positive, which suggests natural selection by increasing genetic variation [55]. In contrast, the D values for *atpD* and *gyrB* were negative, possibly indicating insertion or deletion of nucleotides in the region. Such insertions/deletions of nucleotides could account for the distant, not-so-close, alignment of test isolates with known *Bradyrhizobium* species in the phylogram.

Isolate groupings based on single gene phylogenies and concatenated phylogeny were compared and found to be interesting. Isolates TUTLl2.20.7, TUTMP18.6 and TUTMP19.10 delineated with *B. daqingense*, *B. ottawaense, B. huanghuaihaiense*, *B. liaoningense*, *B. japonicum* and *B. diazoefficiens* in the *nifH* phylogeny, whereas in the concatenated *atpD* + *gyrB* gene phylogeney, they failed to group with any reference strains, thus indicating a lack of correlation between the core and symbiotic gene phylogeny [8]. The results further revealed that the test soybean isolates clustered with the type/reference strains from the genus *Bradyrhizobium*, with *B. japonicum* and *B. diazoefficiens* dominating in all phylogenetic trees. These findings are consistent with other reports which showed that *B. japonicum* is the most dominant soybean microsymbiont in some soils [33], [71].

In the concatenated *atpD* + *gyrB* gene phylogeny, some of the isolates trapped from soils without cropping history grouped together with *B. japonicum* and *B. diazoefficiens*, while some isolates (TUTLl2.20.7, TUTMP19.10, TUTMP18.6) trapped from soil stood alone without any reference strains (Cluster II); this suggests that our bacterial isolates are very diverse, and probably related to different phylogenetic groups of *Bradyrhizobium*. There is therefore the strong possibility that some of these soybean-nodulating bradyrhizobia in South African soils, are unique and novel, especially those trapped from soils without any history of cultivation (e.g. TUTLl2.20.7, TUTMP19.10, TUTMP18.6). But to ascertain that would require further studies to unravel their uniqueness. It was however also interesting to note the grouping of inoculant strains isolated from farmers’ fields specifically with *B. japonicum* and *B. diazoefficiens*.

In conclusion, rhizobial isolates from this study exhibited varying phenotypic, physiological and molecular characteristics, which clearly indicated that the bacterial symbionts isolated from uncultivated soils and from farmers’ fields in Mpumalanga, Limpopo and Gauteng Provinces of South Africa consisted of different bradyrhizobia. These isolates, especially those trapped from soils without any history of cultivation, should be further studied to detail their identity and diversity, while identifying microsymbionts with greater N_2_-fixing ability for use as inoculants for soybean cultivation in Africa.

## Footnotes

Appendix A Supplementary data associated with this article can be found, in the online version, at http://dx.doi.org/10.1016/j.syapm.2016.05.009.
