# Supplementary material for: Microsymbiont diversity and phylogeny of native bradyrhizobia associated with soybean (Glycine max L. Merr.) nodulation in South African soils
Source: Syst Appl Microbiol. 2016 Jul;39(5):336–44. doi: 10.1016/j.syapm.2016.05.009 (PMC4958686; doi:10.1016/j.syapm.2016.05.009)
Supplement: Supplementary file 1 [file mmc1.pdf]

Microsymbiont diversity and phylogeny of native bradyrhizobia associated with soybean (*Glycine max* L. Merr.) nodulation in South African soils

Judith Naamala[1], Sanjay K Jaiswal[2], Felix D Dakora[2]

Table S1

Site description and diversity index of collected soybean bradyrhizobial isolates.

S. No.	Province	No. of sites sampled	Isolates designation	number of isolates	Shannon diversity index (H')	Climatic conditions
1.	Mpumlanga	19	TUTMP	60 (1-60) [(1-54) obtained from farmer fields' nodules; (55-60) from glass house test]	1.47	Rain fall 620 mm (Lowveld)- 878 mm (Highveld), 1-35 ^{o}C temperature annually
2.	Limpopo	7	TUTLI	38 (63-100) glass house	1.37	Rain fall 788 mm, temperature 4-45 ^{o}C annually
3.	Gauteng	1	TUTGP	2 (61-62) glass house	0.69	Rain fall 683 mm, temperature 5-30 ^{o}C annually

Table S2
Primers and PCR temperature profiles used for DNA amplification.

Gene name	Primer Sequence (5' – 3')	Temperature profile	References
16S rDNA	fD1 (9-29)AGAGTTTGATCCTGGCTCAG<br>rD1 (1474-1494)CTTAAGGAGGTGATCCAGCC	5 min 95 °C, 35 X (1 min 95 °C, 1 min 55 °C, 1 min 72 °C), 10 min 72°C	[38]
*nif*H	28f-TACGGNAARGGSGGNATCGGCAA<br>809r-AGCATGTCYTCSAGYYTCNTCCA	5 min 94 °C, 20 X [30 s 94 °C, 30 s 65 °C (-0.5 °C / cycle), 90 s 72 °C],<br>25 X (30 s 94 °C, 30 s 65 °C, 90 s 72 °C), 10 min 72 °C	[43]
*gln*ll	13f-AAGCTCGAGTACATCTGGCTCGACGG<br>681r-SGAGCCGTTCCAGTCGGTGGTGTCG	2 min 95 °C, 35 X (45 s 95 °C, 30 s 65 °C, 90 s 72 °C), 10 min 72 °C	[52]
*gyr*B	343f-TTCGACCAGAAYTCCTAYAAGG<br>1043r-AGCTTGTCCTTSGTCTGCG	10 min 95 °C, 35 X (30 s 94 °C, 30 s 58 °C, 1 min 72 °C), 10 min 72 °C	[36]
*atp*D	189f- TCTGGTCCGYGGCCAGGAAG<br>804r-CGACACTTCCGARCCSGCCTG	2 min 95 °C, 35 X (45 s 95 °C, 30 s 65 °C, 90 s 72 °C), 10 min 72 °C	[52]

Table S3
Accession numbers of the gene sequences of the soybean nodulating isolates used for the phylogenetic study

**Strain**	**16S rDNA**	***atpD***	***gyrB***	***glnII***	***nifH***
TUTMP16.11	KT148835	KT027870	KT027910	KT027888	KT027851
TUTMP18.6	KT148836	KT027871	KT027911	KT027889	KT027852
TUTMP18.11	KT148837	KT027876	KT027912	KT027890	KT027853
TUTMP19.10		KT027877	KT027913	KT027891	KT027854
TUTLl2.20.6	KT729525	KT027878	KT027914	KT027893	KT027855
TUTLl2.10.7	KT729524	KT027879	KT027915	KT027894	KT027856
TUTLl4.10.5b		KT027880	KT027916	KT027895	KT027857
TUTLl4.10.7	KT729526	KT027881	KT027917	KT027896	KT027858
TUTLl4.10.9	KT729522	KT027882	KT027918	KT027897	KT027859
TUTLl7.10.3		KT027883		KT027898	KT027860
TUTLl8.20.5		KT027884	KT027919	KT027903	KT027861
TUTMP2.9	KT148839	KT027885	KT027920	KT027899	KT027862
TUTMP7.7	KT148840	KT027872	KT027921	KT027900	KT027863
TUTMP9.7	KT148841	KT027873	KT027922	KT027901	KT027864
TUTMP12.13		KT027875	KT027924	KT027902	KT027865
TUTMP14.10.3b			KT027926		KT027866
TUTGP10.10.2	KT148838			KT027892	KT027867
TUTLl2.20.7	KT729523	KT351629	KT027927	KT027906	KT027868
TUTMP10.6		KT027886		KT027904	KT027869
TUTMP11.7		KT027874	KT027923	KT027905	
TUTMP14.20.2		KT027887	KT027925	KT027909	
TUTMP17.10.2				KT027908	
TUTMP14.10.3		KT351628	KT027928	KT027907	

Table S4

Phenotypic and 16S rDNA-RFLP characteristics of soybean nodulating bradyrhizobia.

Number used in RFLP-dendrogram	Isolate designation	Field history	Restriction patterns (MspI/*Rsa*I/*Hae*III)	Reaction to BTB	Colony appeared on YMA plate (days)	Colony shape
1	TUTMP1.7	Uninoculated	GFA	Blue	10	Oval
2	TUTMP1.10	Uninoculated	NFE	Blue	9	Domed
3	TUTMP2.9	Inoc. *(B. japonicum)*	GDR	Blue	9	Domed
4	TUTMP2.11	Inoc. *(B. japonicum)*	KGD	Blue	10	Domed
5	TUTMP2.13	Inoc. *(B. japonicum)*	NLE	Blue	10	Domed
6	TUTMP3.7	Uninoculated	MGS	Blue	9	Domed
7	TUTMP3.13	Uninoculated	GDD	Blue	12	Flat
8	TUTMP4.2	Uninoculated	EF-	Blue	7	Domed
9	TUTMP4.13	Uninoculated	GDR	Blue	13	Flat
10	TUTMP5.1	Inoc. *(B. japonicum)*	BBA	Blue	9	Domed
11	TUTMP5.2	Inoc. *(B. japonicum)*	BIA	Blue	8	Domed
12	TUTMP5.3	Inoc. *(B. japonicum)*	NLE	Blue	8	Domed
13	TUTMP5.4a	Inoc. *(B. japonicum)*	BBR	Blue	9	Domed
14	TUTMP5.4b	Inoc. *(B. japonicum)*	BBA	Blue	12	Domed
15	TUTMP5.5	Inoc. *(B. japonicum)*	EDD	Blue	5	Domed
16	TUTMP5.6	Inoc. *(B. japonicum)*	NLE	Blue	7	Domed
17	TUTMP5.7	Inoc. *(B. japonicum)*	GDD	Blue	14	Domed
18	TUTMP5.12	Inoc. *(B. japonicum)*	BBA	Blue	9	Domed
19	TUTMP5.16	Inoc. *(B. japonicum)*	NLA	Yellow	11	Flat
20	TUTMP6.1	Inoc. *(B. japonicum)*	LLL	Blue	9	Domed
21	TUTMP6.4	Inoc. *(B. japonicum)*	GDD	Blue	12	Domed
22	TUTMP6.8	Inoc. *(B. japonicum)*	--D	Blue	9	Flat
23	TUTMP6.20	Inoc. *(B. japonicum)*	NFG	Blue	7	Domed
24	TUTMP7.7	Inoc. *(B. japonicum)*	CJC	Blue	10	Domed
25	TUTMP8.7	Inoc. *(B. japonicum)*	BBR	Blue	12	Domed
26	TUTMP8.9	Inoc. *(B. japonicum)*	LLG	Blue	6	Domed
27	TUTMP8.14	Inoc. *(B. japonicum)*	LKI	Blue	9	Conical
28	TUTMP9.3	Uninoculated	GDA	Blue	10	Domed
29	TUTMP9.5	Uninoculated	NLE	Blue	9	Domed
30	TUTMP9.7	Uninoculated	BBA	Blue	9	Domed
31	TUTMP9.11	Uninoculated	OOS	Blue	12	Domed
32	TUTMP10.6	Inoc. *(B. japonicum)*	GDR	Blue	14	Domed
33	TUTMP10.7	Inoc. *(B. japonicum)*	BBA	Blue	9	Domed
34	TUTMP10.8	Inoc. *(B. japonicum)*	NFG	Blue	6	Domed
35	TUTMP10.10	Inoc. *(B. japonicum)*	NLE	Blue	10	Domed
36	TUTMP10.11	Inoc. *(B. japonicum)*	NLE	Blue	13	Domed

37	TUTMP11.1	Inoc. *(B. japonicum)*	NFE	Yellow	10	Domed
38	TUTMP11.3	Inoc. *(B. japonicum)*	GDD	Blue	12	Domed
39	TUTMP11.7	Inoc. *(B. japonicum)*	GDR	Blue	8	Domed
40	TUTMP11.8	Inoc. *(B. japonicum)*	NLE	Blue	10	Domed
41	TUTMP11.9	Inoc. *(B. japonicum)*	BBR	Blue	8	Domed
42	TUTMP11.13	Inoc. *(B. japonicum)*	BFD	Blue	8	Domed
43	TUTMP11.14	Inoc. *(B. japonicum)*	NFE	Blue	12	Domed
44	TUTMP12.13	Inoc. *(B. japonicum)*	GDD	Blue	9	Domed
45	TUTMP15.3	Uninoculated	ELF	Blue	5	Domed
46	TUTMP15.11	Uninoculated	BBA	Blue	12	Domed
47	TUTMP16.2	Uninoculated	NLF	Yellow	3	Conical
48	TUTMP16.11	Uninoculated	LFG	Blue	10	Domed
49	TUTMP17.15	Uninoculated	GDR	Blue	9	Domed
50	TUTMP18.6	Uninoculated	NLE	Blue	8	Domed
51	TUTMP18.11	Uninoculated	GDD	Blue	12	Domed
52	TUTMP19.10	Uninoculated	LLG	Yellow	8	Domed
53	TUTMP20.8	Uninoculated	GDA	Blue	10	Conical
54	TUTMP20.18	Uninoculated	MGA	Blue	12	Flat
55	TUTMP14.10.1	Uninoculated	PDI	Yellow	4	Domed
56	TUTMP14.20.2	Uninoculated	EDI	Blue	10	Domed
57	TUTMP14.10.3	Uninoculated	EDI	Yellow	2	Domed
58	TUTMP14.10.3b	Uninoculated	HDM	Yellow	2	Domed
59	TUTMP17.10.1	Uninoculated	DBB	Blue	9	Domed
60	TUTMP17.10.2	Uninoculated	DBQ	Yellow	8	Domed
61	TUTGP10.10.1	Uninoculated	CCN	Blue	2	Domed
62	TUTGP10.10.2	Uninoculated	--A	Blue	7	Domed
63	TUTLI1.10.3	Uninoculated	JHH	Yellow	2	Domed
64	TUTLI1.10.3b	Uninoculated	FEJ	Blue	6	Domed
65	TUTLI1.10.4	Uninoculated	PGC	Yellow	2	Domed
66	TUTLI2.10.1	Uninoculated	AAA	Blue	6	Domed
67	TUTLI2.20.1	Uninoculated	AAA	Blue	6	Conical
68	TUTLI2.20.5	Uninoculated	AA-	Blue	6	Domed
69	TUTLI2.20.6	Uninoculated	BBA	Blue	8	Conical
70	TUTLI2.20.7	Uninoculated	-AA	Yellow	2	Domed
71	TUTLI2.10.7	Uninoculated	BBA	Blue	6	Domed
72	TUTLI2.10.7b	Uninoculated	BBA	Blue	7	Domed
73	TUTLI3.10.3	Uninoculated	AAM	Blue	10	Domed
74	TUTLI4.20.3	Uninoculated	AAA	Blue	10	Domed
75	TUTLI4.20.4	Uninoculated	GDD	Blue	6	Domed
76	TUTLI4.10.5	Uninoculated	BBA	Blue	8	Domed
77	TUTLI4.10.5b	Uninoculated	LEJ	Blue	8	Domed
78	TUTLI4.20.5	Uninoculated	GHP	Yellow	2	Domed
79	TUTLI4.10.6	Uninoculated	--A	Blue	6	Domed

80	TUTLI4.10.7	Uninoculated	AAA	Blue	6	Domed
81	TUTLI4.10.9	Uninoculated	AAA	Blue	6	Domed
82	TUTLI4.10.9b	Uninoculated	AAA	Blue	5	Domed
83	TUTLI4.20.10	Uninoculated	BBA	Blue	8	Domed
84	TUTLI4.20.11	Uninoculated	GDD	Blue	9	Domed
85	TUTLI4.10.12	Uninoculated	AAA	Blue	7	Domed
86	TUTLI4.20.16	Uninoculated	KHO	Yellow	3	Domed
87	TUTLI7.10.3	Uninoculated	GDD	Blue	9	Domed
88	TUTLI7.10.4	Uninoculated	-AA	Blue	10	Domed
89	TUTLI710.6	Uninoculated	NBA	Blue	8	Domed
90	TUTLI7.10.7	Uninoculated	BBA	Blue	6	Domed
91	TUTLI8.10.1	Uninoculated	BBA	Blue	4	Domed
92	TUTLI8.20.1	Uninoculated	DBB	Yellow	3	Domed
93	TUTLI8.10.3	Uninoculated	AAA	Blue	8	Domed
94	TUTLI8.20.4	Uninoculated	GDD	Blue	13	Domed
95	TUTLI8.20.5	Uninoculated	IGK	Yellow	3	Flat
96	TUTLI8.20.5b	Uninoculated	IGK	Yellow	2	Domed
97	TUTLI8.10.10	Uninoculated	BBA	Blue	10	Oval
98	TUTLI9.20.1	Uninoculated	CCN	Yellow	2	Domed
99	TUTLI9.20.9	Uninoculated	EDI	Blue	6	Domed
100	TUTLI9.20.9b	Uninoculated	EDI	Blue	6	Domed

Table S5

Information of nucleotide sequences participated in phylogenetic analysis

Locus	No. of strains used for tree construction	Nucleotide sequence information				Total*	Frequency T/C/A/G (%)
		Cnoserved (C)	Variables (V)	Parsimony-informative (Pi)	Singleton (S)		
*atp*D	48	248 (65.26)	132 (34.24)	75 (19.74)	57 (15)	380	19.5/31.3/15.5/33.6
*gln*II	57	184 (61.74)	114 (38.26)	96 (32.21)	18 (6.4)	298	17.1/30.4/17.6/35.0
*gyr*B	48	251 (54.57)	209 (45.43)	120 (26.1)	89 (19.25)	460	19.8/31.0/15.9/33.3
Concatenated (*atp*D+ *gyr*B)	41	509 (60.6)	331 (39.4)	170 (20.24)	161 (19.17)	840	19.6/31.2/15.7/33.5
*nif*H	47	336(63.64)	192(36.36)	147(27.84)	45(8.52)	528	21.8/32.6/19.7/25.9

*number of sites used for tree construction

Table S6
Tajimas' neutral mutation test of all test loci

**Nucleotide information**	**Locus**				
	atpD	***glnII***	***gyrB***	***atpD+gyrB***	***nifH***
m	48	57	48	41	47
S	127	114	208	324	192
P_s	0.345	0.382	0.453	0.391	0.363
$\boldsymbol{\Theta}$	0.078	0.083	0.102	0.092	0.082
$\boldsymbol{\pi}$	0.057	0.099	0.078	0.065	0.102
D	-0.956	0.663	-0.865	-1.085	0.871

m = number of sequences used, *S* = Number of segregating sites, $p_s = S/n$, *n* = total number of sites, $\Theta = p_s/a_1$,
π = nucleotide diversity, and *D* is the Tajima neutrality test

Table S7

Concatenated (*atpD+gyrB*) nucleotide sequence identity (%) of soybean bradyrhizobial isolates with type strains of *Bradyrhizobium*

Strains	1	2	3	4	5	6	7	8	9	10	11	12	13	14	15	16	17	18	19	20	21	22	23	24	25	26	27	28	29	30	31	32	33	34	35	36	37	38	39	40	41
Bradyrhizobium betae (1)	ID																																								
Bradyrhizobium canariense (2)	94.7	ID																																							
Bradyrhizobium daqingense (3)	93.2	93.2	ID																																						
Bradyrhizobiumdenitrificans (4)	90.9	90.2	90.3	ID																																					
Bradyrhizobium diazoefficiens (5)	94.8	94.8	95.1	92.1	ID																																				
Bradyrhizobiumelkanii (6)	92.6	92.8	92.9	90.9	95.0	ID																																			
Bradyrhizobium enbrapense (7)	91.9	92.2	92.6	90.8	93.9	96.0	ID																																		
Bradyrhizobium ganzhouense (8)	93.9	94.0	93.2	89.7	95.3	92.9	92.3	ID																																	
Bradyrhizobium guangdongense (9)	93.6	93.5	93.9	91.7	95.4	93.8	92.9	93.4	ID																																
Bradyrhizobium guangxiense (10)	92.8	93.4	95.0	91.1	94.8	93.0	91.7	92.7	94.2	ID																															
Bradyrhizobium huanghuaihaiense(11)	93.6	93.8	95.9	91.1	96.5	94.0	93.2	94.7	94.2	94.6	ID																														
Bradyrhizobium japonicum (12)	94.1	94.7	94.1	91.4	96.5	94.6	93.4	94.4	95.3	94.0	95.0	ID																													
Bradyrhizobium jicamae (13)	91.1	93.0	91.6	89.7	93.6	93.6	92.5	92.2	92.7	92.6	93.2	93.4	ID																												
Bradyrhizobium lablabi (14)	90.7	92.3	91.3	89.5	92.5	92.7	91.3	91.5	92.7	92.0	91.9	92.3	96.1	ID																											
Bradyrhizobium liaoningense (15)	94.0	94.4	94.1	91.5	95.3	93.3	92.6	93.8	94.5	94.4	94.7	94.7	92.8	92.0	ID																										
Bradyrhizobium ottawaense (16)	93.9	92.9	93.2	89.6	95.8	93.4	92.1	93.9	93.4	93.0	94.7	94.6	92.3	91.3	94.0	ID																									
Bradyrhizobium pachyrhizi (17)	92.6	92.7	92.6	90.9	94.6	98.2	95.9	93.5	93.5	92.6	93.6	94.0	93.0	92.8	93.0	92.7	ID																								
Bradyrhizobium paxllaeri (18)	89.5	92.3	90.5	89.5	92.0	91.5	91.6	91.0	91.3	91.4	91.4	91.3	95.8	95.9	91.5	90.0	92.1	ID																							
Bradyrhizobium retamae (19)	88.4	91.0	89.8	88.4	90.7	90.8	89.5	89.1	89.4	89.6	90.2	90.7	92.3	92.6	89.8	89.1	90.7	92.1	ID																						
Bradyrhizobium rifense (20)	94.2	95.5	93.6	90.4	95.5	92.6	92.0	95.9	93.5	94.1	95.3	94.1	92.8	91.3	94.1	93.6	92.9	91.4	89.7	ID																					
Bradyrhizobium tropiciagri (21)	92.3	92.7	92.5	90.8	94.7	96.1	96.3	92.7	93.8	92.7	93.5	93.9	93.3	92.3	92.6	92.3	96.5	91.5	89.8	92.7	ID																				
Bradyrhizobium yuanmingense (22)	92.7	92.9	94.5	91.9	94.7	94.0	93.2	92.7	93.5	94.5	94.7	94.5	92.0	90.8	94.0	92.9	93.5	90.5	89.1	93.2	93.9	ID																			
Rhizobium lusitanum P1-7 (23)	73.6	74.6	74.5	75.3	74.1	74.7	74.8	74.1	73.5	75.0	74.5	74.0	74.6	74.5	74.4	73.4	75.0	74.0	72.8	74.6	75.1	75.0	ID																		
TUTLl2.10.7 (24)	94.4	94.6	94.5	91.9	97.3	94.2	93.0	94.5	96.0	94.7	95.5	98.3	93.0	92.1	94.8	94.2	93.9	91.1	90.2	94.6	94.0	94.1	74.5	ID																	
TUTLl2.20.6 (25)	94.4	94.6	94.5	91.9	97.3	94.2	93.0	94.5	96.0	94.7	95.5	98.3	93.0	92.1	94.8	94.2	93.9	91.1	90.2	94.6	94.0	94.1	74.5	100	ID																
TUTLl2.20.7 (26)	94.0	93.9	95.4	91.7	96.6	94.4	93.5	94.2	95.7	94.7	96.3	96.1	93.4	92.5	94.5	94.2	94.0	91.4	90.2	94.7	94.5	95.0	74.2	97.0	97.0	ID															
TUTLl4.10.5b (27)	94.8	94.8	95.1	92.1	100	95.0	93.9	95.3	95.4	94.8	96.5	96.5	93.6	92.5	95.3	95.8	94.6	92.0	90.7	95.5	94.7	94.7	74.1	97.3	97.3	96.6	ID														
TUTLl4.10.7 (28)	94.8	94.8	95.1	92.1	100	95.0	93.9	95.3	95.4	94.8	96.5	96.5	93.6	92.5	95.3	95.8	94.6	92.0	90.7	95.5	94.7	94.7	74.1	97.3	97.3	96.6	100	ID													
TUTLl4.10.9 (29)	94.2	94.8	94.5	91.7	99.1	95.0	94.1	95.4	94.8	94.1	96.0	97.3	93.9	92.6	95.1	95.5	94.6	92.2	90.8	95.1	94.7	94.7	74.1	96.5	96.5	95.8	99.1	99.1	ID												
TUTLl8.20.5 (30)	94.4	94.6	94.5	91.9	97.3	94.2	93.0	94.5	96.0	94.7	95.5	98.3	93.0	92.1	94.8	94.2	93.9	91.1	90.2	94.6	94.0	94.1	74.5	100	100	97.0	97.3	97.3	96.5	ID											
TUTMP11.7 (31)	93.0	93.5	93.6	91.3	97.5	95.4	94.4	93.9	93.8	93.2	95.2	95.1	92.3	91.6	94.5	94.2	95.7	91.3	90.3	93.6	94.6	94.2	74.4	94.8	94.8	94.1	97.5	97.5	97.5	94.8	ID										
TUTMP12.13 (32)	93.0	94.7	93.4	91.5	95.3	93.3	92.7	93.3	94.2	94.1	94.5	97.1	93.5	92.3	94.6	92.7	93.6	92.0	90.8	94.0	92.9	93.5	74.8	97.9	97.9	95.0	95.3	95.3	95.3	97.9	94.7	ID									
TUTMP14.10.3 (33)	94.8	94.8	95.1	92.1	100	95.0	93.9	95.3	95.4	94.8	96.5	96.5	93.6	92.5	95.3	95.8	94.6	92.0	90.7	95.5	94.7	94.7	74.1	97.3	97.3	96.6	100	100	99.1	97.3	97.5	95.3	ID								
TUTMP14.20.2 (34)	93.8	93.4	95.3	92.2	96.9	94.6	93.3	93.8	95.0	95.5	95.9	95.3	93.4	92.6	94.2	94.4	94.4	91.9	90.2	94.7	94.5	95.3	74.6	96.3	96.3	96.7	96.9	96.9	96.0	96.3	94.4	94.2	96.9	ID							
TUTMP16.11 (35)	94.8	94.8	95.1	92.1	100	95.0	93.9	95.3	95.4	94.8	96.5	96.5	93.6	92.5	95.3	95.8	94.6	92.0	90.7	95.5	94.7	94.7	74.1	97.3	97.3	96.6	100	100	99.1	97.3	97.5	95.3	100.0	96.9	ID						
TUTMP18.11 (36)	94.8	94.8	95.1	92.1	100	95.0	93.9	95.3	95.4	94.8	96.5	96.5	93.6	92.5	95.3	95.8	94.6	92.0	90.7	95.5	94.7	94.7	74.1	97.3	97.3	96.6	100	100	99.1	97.3	97.5	95.3	100.0	96.9	100	ID					
TUTMP18.6 (37)	93.9	93.6	94.8	92.6	97.0	93.8	93.2	94.0	96.0	94.8	95.9	96.4	93.2	92.2	95.5	94.2	93.4	91.1	90.1	94.5	94.4	95.3	74.0	97.3	97.3	97.5	97.0	97.0	96.1	97.3	94.5	95.3	97.0	96.6	97.0	97.0	ID				
TUTMP19.10 (38)	93.4	93.9	94.8	91.4	95.8	94.4	93.8	94.4	95.1	94.0	95.8	97.0	93.6	92.6	94.2	94.0	94.0	91.6	90.3	94.2	94.5	95.0	74.2	96.1	96.1	99.1	95.8	95.8	96.6	96.1	94.1	95.0	95.8	95.9	95.8	95.8	96.6				
TUTMP2.9 (39)	94.0	94.5	94.0	91.6	98.4	94.7	94.0	94.1	94.1	94.0	95.3	95.8	93.8	92.3	94.6	95.2	94.4	92.1	90.5	94.5	94.4	95.3	74.2	95.8	95.8	95.1	98.4	98.4	98.4	95.8	97.3	95.7	98.4	95.3	98.4	98.4	95.4	95.1			
TUTMP7.7 (40)	93.5	95.0	94.0	91.7	97.9	94.0	93.5	94.1	93.6	94.2	95.4	95.3	94.1	92.7	95.1	94.2	94.4	92.8	91.3	95.0	93.6	94.1	74.5	95.3	95.3	94.6	97.9	97.9	97.9	95.3	97.3	97.3	97.9	94.8	97.9	97.9	95.0	94.6	98.3		
TUTMP9.7 (41)	93.4	94.2	93.8	91.9	97.7	94.7	93.8	94.0	93.9	93.8	95.2	95.4	93.2	92.0	94.6	94.2	94.1	91.6	90.7	94.4	94.0	94.1	74.5	95.1	95.1	94.4	97.7	97.7	97.8	95.1	97.3	95.2	97.7	94.6	97.7	97.7	94.7	94.5	97.8	97.8	ID

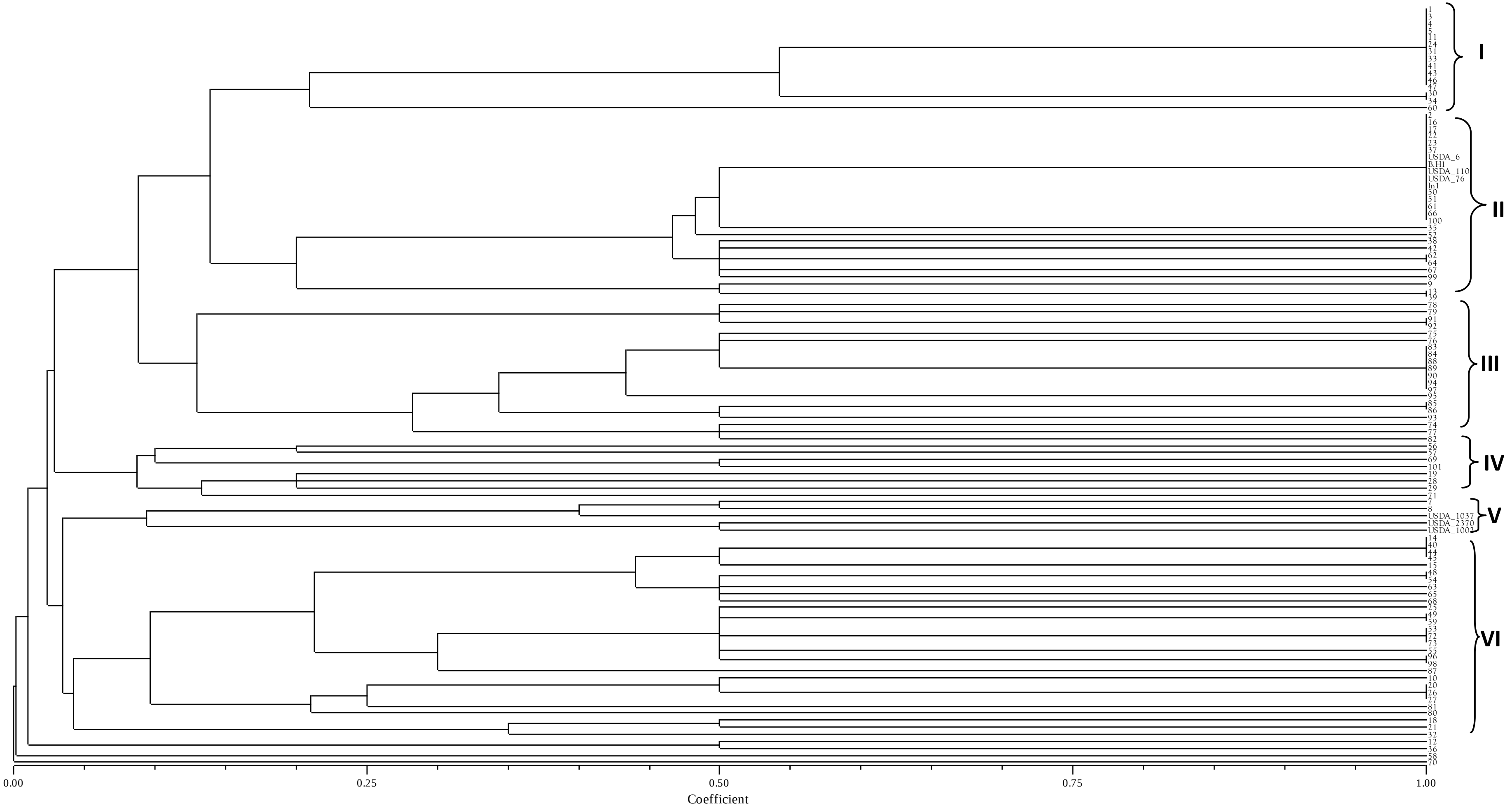

Fig. S1: Dendrogram generated from 16S- rDNA-RFLP banding pattern data of soybean nodulating bradyrhizobial isolates

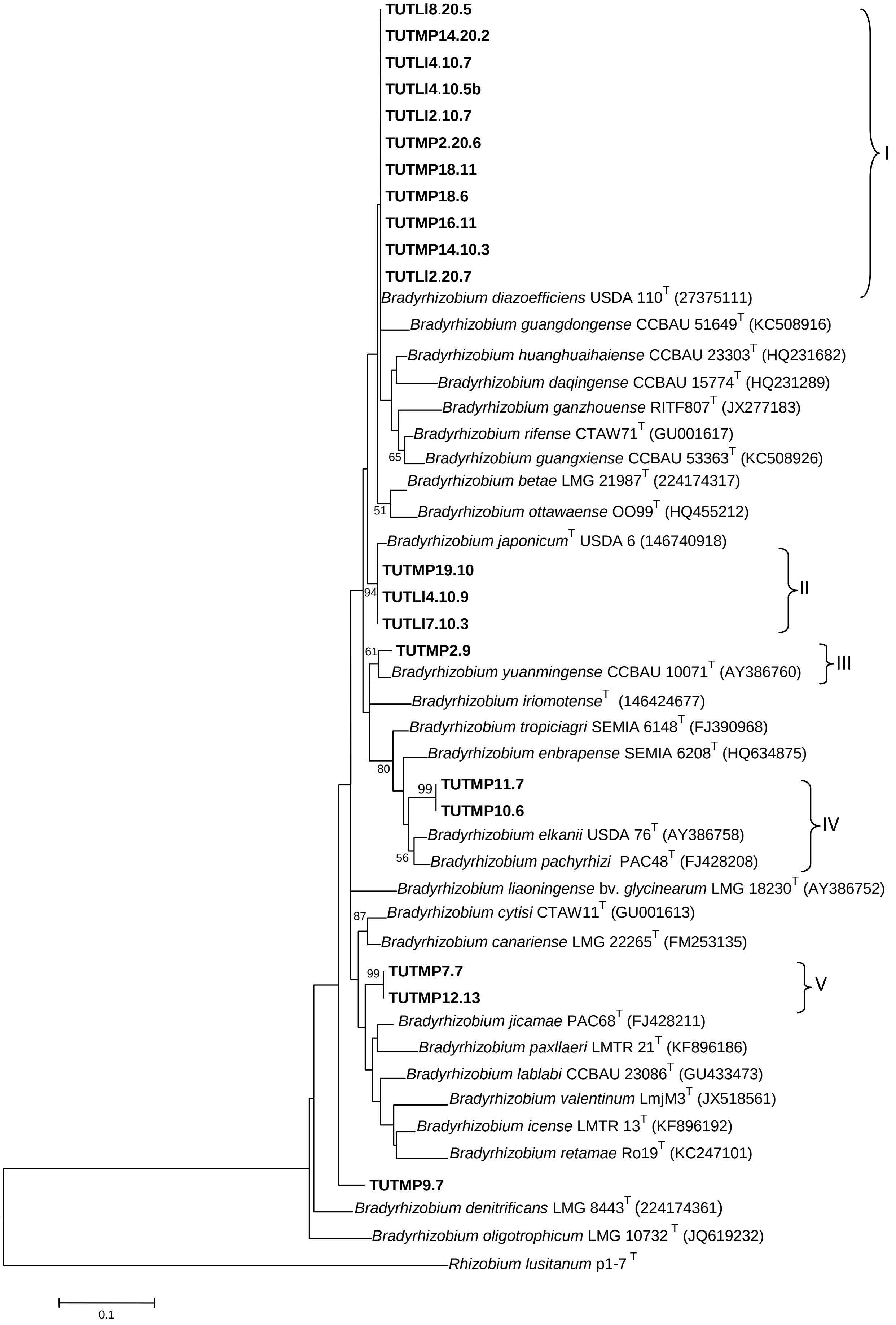

Fig. S2: Phylogenetic tree based on *atp*D sequences generated by Maximum-Likelihood algorithm. Bootstrap values (1000 replicates) are indicated above the branches.

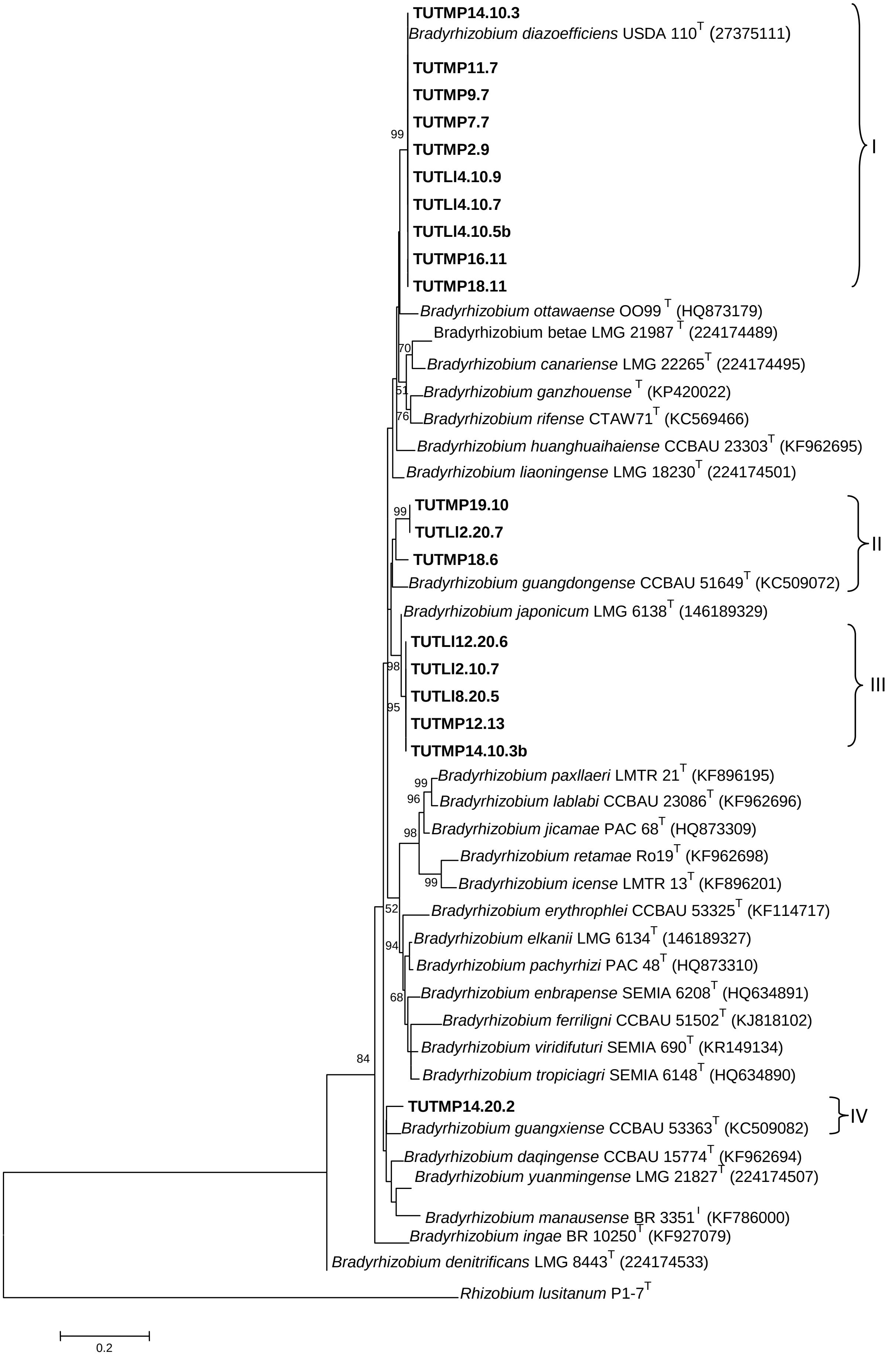

Fig. S3: Phylogenetic tree based on *gyrB* sequences generated by Maximum-Likelihood algorithm. Bootstrap values (1000 replicates) are indicated above the branches.
